# Hypoxia induced the differentiation of Tbx18-positive epicardial cells to CoSMCs

**DOI:** 10.1038/srep30468

**Published:** 2016-07-26

**Authors:** Xiaodong Jing, Yulin Gao, Songlin Xiao, Qin Qin, Xiaoming Wei, Yuling Yan, Ling Wu, Songbai Deng, Jianlin Du, Yajie Liu, Qiang She

**Affiliations:** 1 Department of Cardiology, The Second Affiliated Hospital of Chongqing Medical University, Chongqing, 400016, China

## Abstract

Understanding the origin and differentiation mechanism of coronary vascular smooth muscle cells (CoSMCs) is very important to cardiovascular biology. The early cardiovascular system is formed in a hypoxic microenvironment, and Tbx18-positive epicardial cells are a source of CoSMCs. However, the effects of hypoxia on the differentiation of Tbx18-positive epicardial cells to CoSMCs and the primary regulatory mechanism are insufficiently understood. Using Tbx18:Cre/R26R^EYFP/LacZ^ fate-tracing mice, we cultured highly purified Tbx18-positive epicardial cells. We further showed that hypoxia induced Tbx18-positive epicardial cells to differentiate into CoSMCs and promoted the epithelial-mesenchymal transition (EMT) process of the cells *in vitro*. The induction of differentiation was primarily achieved via the hypoxia inducible factor-1α (HIF-1α)-mediated effects exerted on Snail. Using a cell migration assay, we showed that hypoxia enhanced the motility of Tbx18-positive epicardial cells. By constructing a hypoxic model of the embryonic epicardium *in vivo*, we showed that hypoxia led to premature *in situ* differentiation of Tbx18-positive epicardial cells to CoSMCs. Furthermore, hypoxia was sufficient to induce Snail expression in Tbx18-positive epicardial cells *in vivo*. Our study suggests that hypoxia intervention was sufficient to induce the differentiation of Tbx18-positive epicardial cells to CoSMCs. Furthermore, this differentiation was achieved primarily via HIF-1α-mediated regulation of Snail.

Coronary vascular smooth muscle cells (CoSMCs) provide structural support for the contraction of coronary vessels and play important roles in the pathophysiological process of cardiovascular disease. Therefore, a study of the origin and differentiation of CoSMCs is of great significance. Research in mammalian embryos indicated that embryonic epicardium is the main source of CoSMCs^1^. The embryonic epicardium is a layer of epithelial tissue, located in the surface of the heart. The embryonic epicardium contains a pool of progenitor cells expressing a variety of progenitor cell markers, including Tbx18, WT1, and Tcf21. Lineage tracing studies have demonstrated that a certain subset of epicardial cells undergo the epithelial-mesenchymal transition (EMT) process to generate a population of epicardium-derived cells (EPDCs) that differentiate to CoSMCs and fibroblasts^2^,^3^,^4^. Tbx18-positive epicardial cells are a subset of progenitor cells that express the T-box transcription factor Tbx18. The T-box family was shown to play a crucial role in the process of embryonic heart development^5^,^6^. Lineage tracing studies using Cre-LoxP technology (Tbx18:Cre) have confirmed that Tbx18-positive epicardial cells are pluripotent progenitor cells, which can differentiate into CoSMCs, fibroblasts, and sinoatrial node cells^7^,^8^,^9^,^10^. The EMT process is the critical mechanism by which epicardial cells migrate into the mesenchyme and differentiate to specified EPDCs. At E9.5, Tbx18-positive proepicardial cells begin to migrate to the outermost layer of the heart and subsequently form the Tbx18-positive epicardial cells on the surface of the heart at E10.5. Next, Tbx18-positive epicardial cells migrate into the heart via the EMT process and differentiate into CoSMCs and fibroblasts. The activation of EMT in different subsets of epicardial cells relies on local molecular and cellular signals. The process of EMT involves a complex regulatory network of multiple signalling molecules^11^,^12^,^13^, and the zinc finger transcription factors Snail and Slug are key regulators of this process^14^,^15^,^16^,^17^. Various signalling pathways and cell types have been shown to regulate the EMT of epicardial cells through the regulation of Snail and Slug^18^,^19^,^20^,^21^. However, the regulatory mechanisms underlying the EMT process of Tbx18-positive epicardial cells and their differentiation to CoSMCs have not yet been totally elucidated.

One important feature of mammalian embryonic development is the hypoxic environment of early embryonic tissue^22^. This condition is primarily attributed to the fact that the diffusion of oxygen cannot meet the demands of the rapid development of embryonic tissue. The formation of the early cardiovascular system occurs in a hypoxic microenvironment^23^. The adaptation of the cardiovascular system to hypoxia was shown to promote the expression of specific genes, which support the formation and development of the coronary circulatory system. The cellular hypoxic response was shown to be regulated by hypoxia inducible factor-1α (HIF-1α)^24^. Research with chicken embryos confirmed the expression of HIF-1α in the early embryonic heart and its association with coronary vascular development^25^. Previous work indicated that the knockout of HIF-1α caused coronary vascular malformations, interstitial cell death, cardiac abnormalities and the death of E11.0 embryos^26^. Presently, the literature has confirmed that Snail contains hypoxia response element (HRE) fragments in its gene promoter, and hypoxia can regulate the expression of Snail via HIF-1α to induce EMT^27^. Therefore, we hypothesized that hypoxia may be one of the mechanisms that can induce the EMT process and the differentiation of Tbx18-positive epicardial cells to CoSMCs.

Genetic lineage tracing techniques, such as the Cre-LoxP system, have been widely used in the field of development. Tbx18:Cre/R26R^EYFP^ and Tbx18:Cre/R26R^Lacz^ fate-mapping mouse models were produced by crossing Tbx18:Cre knock-in mice with the Cre reporter mice R26R^EYFP^ and R26R^Lacz^ to monitor Tbx18-positive epicardial cells^9^. These two fate-mapping models are reliable methods to observe the fate of Tbx18-positive cardiac progenitor cells *in vivo* and *in vitro*. In our research, to visualize Tbx18-positive epicardial cells and their differentiation, we identified the Tbx18 fate-tracing model by genotype analysis and further cultured Tbx18-positive epicardial cells *in vitro*. The β-gal staining of Tbx18:Cre/R26R^Lacz^ mice and the YFP fluorescence of Tbx18:Cre/R26R^EYFP^ mice both permit the visualization of Tbx18-positive cells *in vivo* and *in vitro*. We further exposed Tbx18-positive epicardial cells to hypoxia and studied the role of hypoxia in their differentiation to CoSMCs and the EMT process.

## Results

### Culture of Tbx18-positive epicardial cells

**Figure 1:**
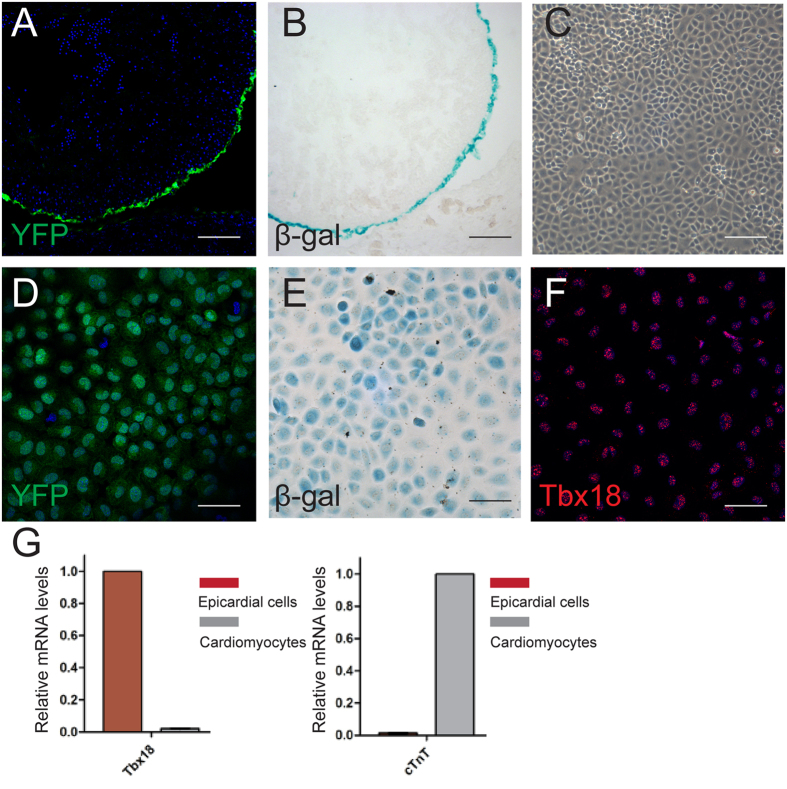
Culture of Tbx18-positive epicardial cells from E11.5 Tbx18:Cre/R26R^EYFP/LacZ^ tracing hearts. (**A**) Direct YFP expression analysis in cryosections of E11.5 Tbx18:Cre/R26R^EYFP^ tracing hearts showed that YFP fluorescence was specifically expressed in the epicardium. (**B**) The β-gal staining of E11.5 hearts from Tbx18:Cre/R26R^Lacz^ embryos showed that the epicardium was β-gal-positive. (**C**) Representative image of Tbx18-positive epicardial cells. (**D**) YFP expression of Tbx18-positive epicardial cells isolated from Tbx18:Cre/R26R^EYFP^ tracing hearts. (**E**) β-gal-positive staining of cells isolated from Tbx18:Cre/R26R^LacZ^ tracing hearts. (**F**) Immunostaining for Tbx18 and DAPI nuclear staining of the cultured epicardial cells. (**G**) qRT-PCR analyses of Tbx18 vs. cTnt (cardiomyocyte-specific marker) expression in Tbx18-positive epicardial cells and cardiomyocytes (p < 0.001). The data are presented as the means ± SD of three experiments, Student’s t test. Scale bars in (**A–C**) are 100 μm and scale bars in (**D–F**) are 50 μm.

To investigate the effects of hypoxia on Tbx18-positive epicardial cells *in vitro*, we first genotyped E11.5 Tbx18:Cre/R26R^EYFP/LacZ^ hearts and cultured Tbx18-positive epicardial cells from the explanted hearts. The epicardium of E11.5 Tbx18:Cre/R26R^EYFP/Lacz^ hearts specifically expressed YFP fluorescence or positive β-gal staining (Fig. 1A,B), which allowed for the visualization of endogenous Tbx18 expression throughout heart development^9^. After 48 h of culture, the cells migrated from the surface of the hearts and displayed an epithelial-like morphology (Fig. 1C). The Tbx18-positive epicardial cells obtained from Tbx18:Cre/R26R^EYFP/Lacz^ hearts also showed YFP fluorescence or stained positive for β-gal separately (Fig. 1D,E). Immunofluorescence staining showed that these cells expressed Tbx18 (Fig. 1F). Furthermore, these cells cultured from the Tbx18-tracing mice expressed high levels of Tbx18 mRNA (Fig. 1G). Meanwhile, the results of staining for a cardiac fibroblast marker (periostin) also confirmed that there was no cardiac fibroblast contamination among the cultured Tbx18-positive epicardial cells (Supplementary Fig. S1). Thus, we established highly pure cultures of Tbx18-positive epicardial cells using Tbx18 genetic lineage tracing mice, and we could further trace the differentiation of these cells by monitoring their YFP fluorescence and β-gal staining.

### Hypoxia induced the differentiation of Tbx18-positive epicardial cells to CoSMCs

**Figure 2:**
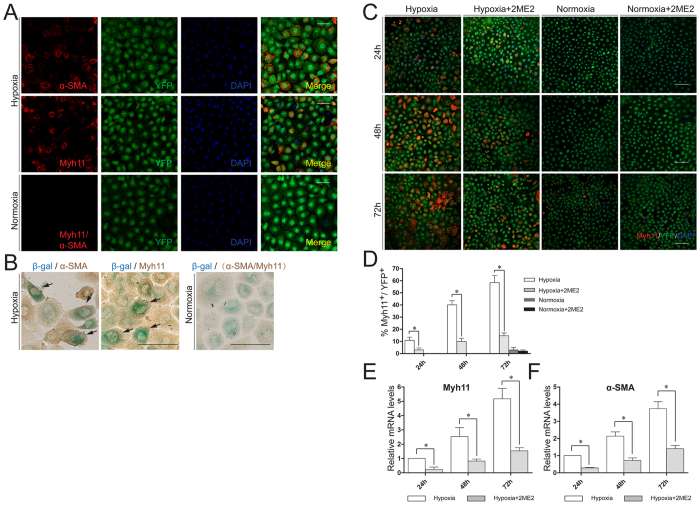
Hypoxia induced the differentiation of Tbx18-positive epicardial cells to CoSMCs. (**A**) Immunofluorescence analyses of Tbx18-positive epicardial cells cultured under hypoxia for 48 h for the expression of α-SMA and myh11. Scale bar is 50 μm. (**B**) Immunohistochemistry for α-SMA and myh11 (brown) and β-gal staining (blue) in Tbx18:Cre/R26R^Lacz^ -tracing epicardial cells after culture under hypoxia for 48 h. Co-staining for α-SMA or myh11 with β-gal produced a dark olive-green colour. Scale bar is 25 μm. (**C**) Tbx18-positive epicardial cells were cultured under hypoxia, hypoxia combined with 2ME2, normoxia, or normoxia combined with 2ME2 for 24 h, 48 h or 72 h. Then, the cells were immunostained for myh11. Scale bar is 100 μm. (**D**) The percentage of myh11+/YFP + cells was quantified by scoring 1,000 cells per group from three independent experiments. The data are shown as the means ± SD. *p < 0.05; Student’s t test for two groups at 24 h and 48 h; one-way ANOVA followed by Tukey test for the four groups at 72 h. (**E,F**) The mRNA expression levels of α-SMA and myh11 were determined in Tbx18-positive epicardial cells cultured under hypoxia or hypoxia combined with 2ME2 for 24 h, 48 h or 72 h by qRT-PCR. GAPDH was used as an internal control, and the mRNA levels in hypoxia groups were used as controls. The data are shown as the means ± SD of three experiments. *p < 0.05; Student’s t test.

Hypoxia is one of the key signals involved in early embryonic cardiovascular development. Cobalt chloride (CoCl_2_) is widely used to artificially mimic hypoxia in cultured cells^28^,^29^. In this study, 200 μmol/L CoCl_2_ was chosen to induce a hypoxic response (Supplementary methods, Supplementary Figs S2A and S3). We treated cells with 200 μmol/L CoCl_2_ to induce hypoxic conditions and investigated the differentiation of Tbx18-positive epicardial cells to CoSMCs under these hypoxic conditions. After culturing under hypoxic conditions for 48 h, a significant increase was observed in the number of Tbx18:Cre/R26R^EYFP^ lineage-traced Tbx18-positive epicardial cells expressing markers of CoSMCs (Fig. 2A). Immunostaining analyses also demonstrated the efficient differentiation of Tbx18:Cre/R26R^Lacz^ lineage-traced Tbx18-positive epicardial cells to CoSMCs (Fig. 2B). These results clearly showed that CoCl_2_-induced hypoxia was sufficient to induce the differentiation of Tbx18-positive epicardial cells to CoSMCs. The extent of differentiation increased with longer hypoxia exposure (Fig. 2C,D). Contraction after stimulation was characteristic of smooth muscle cells. We next sought to examine whether these differentiated CoSMCs could contract in response to carbachol, a muscarinic agonist. The Tbx18-positive epicardial cells were cultured in hypoxia for 72 h and then imaged before and after 1 mmol/L carbachol treatment. The results showed that a portion of hypoxia-induced cells were shortened after 5 min of carbachol treatment (Supplementary Fig. S4).

The cellular hypoxic response was mainly regulated by hypoxia inducible factor-1α (HIF-1α)^24^. To confirm the role of HIF-1α in the differentiation of Tbx18-positive epicardial cells, we blocked the nuclear accumulation of HIF-1α with the HIF-1α inhibitor 2-Methoxyestradiol (2ME2)^30^ at a concentration of 50 μmol/L (Supplementary methods, Supplementary Figs S2B and S3). Subsequently, the differentiation rates significantly decreased after the inhibition of HIF-1α (Fig. 2D, 10.8% ± 2.63% vs. 3% ± 1.61%, 24 h, *p* = 0.012; 40.06% ± 3.42% vs. 9.87% ± 2.71%, 48 h, *p* < 0.001; 58.53% ± 5.78% vs. 14.63% ± 1.34%, 72 h, *p* < 0.001). qRT-PCR and Western blot assays were further used to determine the expression of CoSMCs markers. The results showed that the mRNA levels of myh11 and α-SMA gradually increased after hypoxia exposure, and these increases were obviously inhibited by blocking HIF-1α (Fig. 2E,F). Meanwhile, the increases of myh11 protein levels after hypoxia exposure were also inhibited by blocking HIF-1α (Supplementary Fig. S5). These results suggested that hypoxia induced Tbx18-positive epicardial cells to differentiate into CoSMCs and that HIF-1α was likely to be a key regulator of this hypoxia-induced differentiation.

### Hypoxia promoted the EMT process of Tbx18-positive epicardial cells

**Figure 3:**
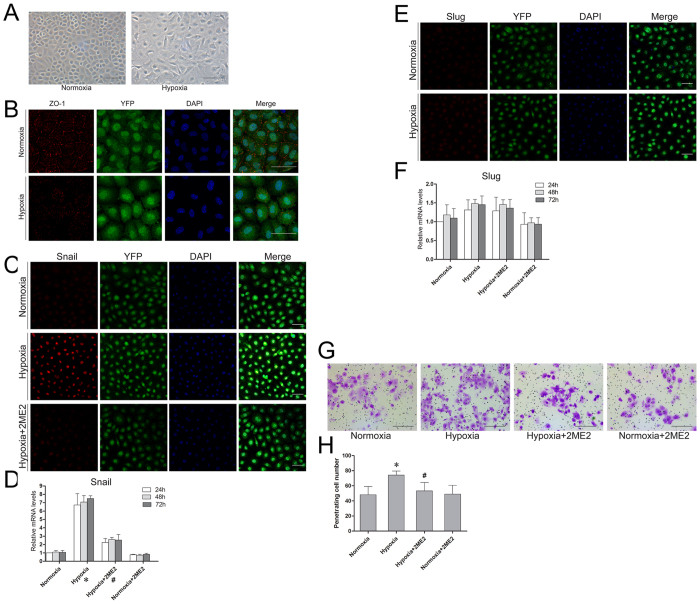
Effects of hypoxia on the EMT process and the migration of Tbx18-positive epicardial cells. (**A**) Representative morphological changes of Tbx18-positive epicardial cells cultured under normoxia and hypoxia. (**B**) Immunostaining for ZO-1 (red) and DAPI nuclear staining (blue) of Tbx18-positive epicardial cells cultured under normoxia and hypoxia for 24 h. (**C**) Immunostaining for Snail (red) and DAPI nuclear staining (blue) of Tbx18-positive epicardial cells cultured under normoxia, hypoxia and hypoxia + 2ME2 for 24 h. (**D,F**) The mRNA expression levels of Snail and Slug were determined under normoxia, hypoxia, hypoxia combined with 2ME2, or normoxia combined with 2ME2 for 24 h, 48 h or 72 h by qRT-PCR. GAPDH was used as an internal control, and the mRNA levels in Tbx18-positive epicardial cells cultured under normoxia were used as controls. The data were shown as the means ± SD of three experiments. *p < 0.05, hypoxia group vs. normoxia group at each time point; ^#^p < 0.05, hypoxia group vs. hypoxia + 2ME2 group at each time point; one-way ANOVA. (**E**) Immunostaining for Slug (red) and DAPI nuclear staining (blue) under normoxia and hypoxia for 24 h. (**G**) Representative images of crystal violet staining in each group. (**H**) The numbers of migrated cells were quantified by counting the cells from 5 random microscopic fields. *p < 0.05, hypoxia group vs. normoxia group; ^#^p < 0.05, hypoxia group vs. hypoxia + 2ME2 group; one-way ANOVA followed by Tukey test. Scale bars in (**A–C**) are 50 μm and scale bars in G are 100 μm.

The EMT process is the main mechanism underlying the differentiation of epicardial cells to mesenchymal cells^2^,^3^,^4^. Therefore, we further investigated the effects of hypoxia on the EMT process in these cells as well as the underlying mechanisms. Under normoxic conditions, Tbx18-positive epicardial cells resembled epithelial-like progenitor cells and maintained a cobblestone-like cell shape (Fig. 3A). After culture under hypoxic conditions for 24 h, the epicardial features of the cells disappeared, and they exhibited a mesenchymal phenotype (Fig. 3A).To further observe this morphological change, we examined the expression of zonula occludens-1 (ZO-1) in these cells. When the cells were cultured under normoxia, ZO-1 was normally expressed at the junctions of the cells in a “linear” arrangement (Fig. 3B). After culture under hypoxic conditions for 24 h, ZO-1 expression was lost from the cell-cell junctions (Fig. 3B). To further confirm whether hypoxia induced the EMT process in Tbx18-positive epicardial cells, we next used Western blot to determine the protein levels of the epicardial markers (E-cadherin and claudin-1) at the three hypoxia time points. The results showed that the expression of E-cadherin and claudin-1 was decreased after the cells were cultured at each time point (Supplementary Fig. S6). The mRNA levels of N-cadherin were also obviously increased after the cells were cultured under hypoxia (Supplementary Fig. S7). Thus, these data indicated that the EMT process in Tbx18-positive epicardial cells was induced by hypoxia intervention.

### Snail was a potential downstream target of HIF-1α

Snail and Slug are major regulators of EMT^14^,^15^,^16^,^17^. We next explored the effects of hypoxia on these EMT regulators in Tbx18-positive epicardial cells and tried to elucidate the downstream factors of HIF-1α. Snail and Slug were both weakly expressed in Tbx18-positive epicardial cells under normoxia (Fig. 3C,E). After the cells were cultured under hypoxia for 24 h, the expression of Snail was greatly increased and primarily located in the nuclei of the cells (Fig. 3C), whereas Slug staining was not significantly altered with hypoxia (Fig. 3E). In addition, hypoxia exposure increased the Snail mRNA transcript levels by nearly 6-fold compared with the levels under normoxia (Fig. 3D). However, the mRNA levels of Slug were not significantly changed by hypoxia exposure (Fig. 3F). We further blocked HIF-1α to observe the changes in Snail expression. The immunofluorescence staining results showed that the increased expression of Snail was significantly inhibited by blocking HIF-1α for 24 h (Fig. 3C). Meanwhile, the hypoxia-induced increased mRNA levels of Snail were also inhibited at 24 h, 48 h and 72 h by blocking HIF-1α, with the altered Snail mRNA levels changing from a 6-fold increase to an approximately 2-fold increase (Fig. 3D). However, the mRNA levels of Slug were not significantly changed by blocking HIF-1α under hypoxic conditions (Fig. 3F). These results indicated that Snail, rather than Slug, was likely to be the downstream target of HIF-1α under hypoxic conditions, and the expression of Snail was presumably directly induced by HIF-1α.

### Knockdown of Snail inhibited the hypoxia-medicated CoSMCs differentiation

Our research has shown that hypoxia induces the CoSMCs differentiation of Tbx18-positive epicardial cells via HIF-1α and also increases the expression of Snail. To determine whether Snail is involved in this hypoxia-induced differentiation, we next performed knockdown experiments for Snail in hypoxic Tbx18-positive epicardial cells. The knockdown efficiency was analysed by qRT-PCR and Western blot. The results showed that after 48 h of siRNA transfection under hypoxia, the expression of Snail was obviously down-regulated at both the mRNA and protein levels compared with the hypoxia group (Supplementary Fig. S8). We further observed the expression changes for α-SMA and myh11 after the knockdown of Snail in hypoxic cells. Our results showed that knockdown of Snail also decreased the mRNA and protein expression of α-SMA and myh11 in these hypoxic cells (Supplementary Fig. S8). Collagen contraction assay was further used to evaluate the contractility of these differentiated CoSMCs. The cells differentiated under hypoxic condition showed a significant increase in collagen gel contraction after treated with carbachol (84.3 ± 3.5% vs. 57.0 ± 4.0%, *p* = 0.001). However, no obvious change in carbachol-induced contraction was observed after the knockdown of Snail in hypoxic cells (Supplementary Fig. S9). Thus, knockdown of Snail in hypoxic Tbx18-positive epicardial cells could inhibit their differentiation to CoSMCs.

### Hypoxia enhanced the migration of Tbx18-positive epicardial cells

Hypoxia intervention and mesenchymal transition were reported to increase cell motility^27^,^31^. A transwell assay was further used in our study to clarify the role of hypoxia and the requirement for HIF-1α in epicardial cell migration. As shown in Fig. 3G,H, hypoxia markedly increased the migration of Tbx18-positive epicardial cells in comparison with normoxia-cultured cells (48.2 ± 10.9, normoxia vs. 74.2 ± 5.4, hypoxia, *p* = 0.001). Moreover, the hypoxia-induced enhanced cell migration was inhibited by blocking HIF-1α (74.2 ± 5.4, hypoxia alone vs. 53.4 ± 11.13, hypoxia combined with 2ME2, *p* = 0.006). These data indicated that hypoxia enhanced the migration of Tbx18-positive epicardial cells in a HIF-1α-dependent manner.

We further constructed an *in vivo* hypoxia model of the embryonic epicardium to explore the effects of hypoxia on the differentiation of Tbx18-positive epicardial cells (Supplementary methods). The hypoxyprobe-1 staining results showed that the epicardium started to become hypoxic after 3 h of maternal hypoxia. Furthermore, the hypoxic region then encompassed the myocardium and ventricular septum after 24 h of maternal hypoxia (Supplementary Fig. S10A). Meanwhile, HIF-1α-expressing Tbx18-positive epicardial cells were also obviously increased after hypoxia intervention (Supplementary Fig. S10B). Thus, we successfully constructed an *in vivo* embryonic epicardium hypoxia model.

### Tbx18-positive epicardial cells differentiated into CoSMCs *in vivo*

**Figure 4:**
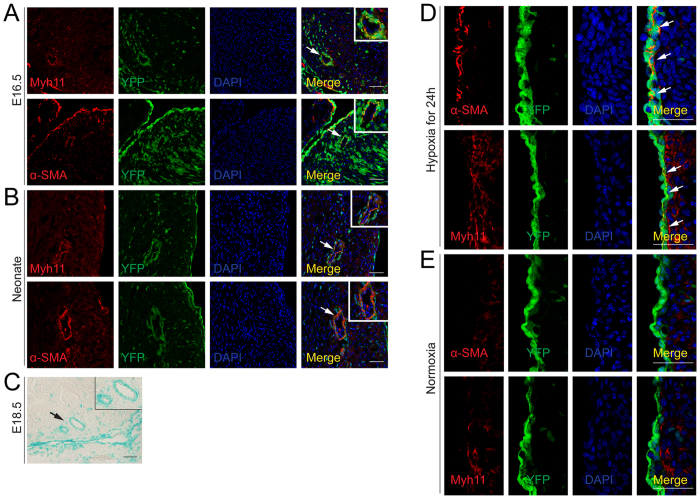
Tbx18-positive epicardial cells differentiated into CoSMCs and hypoxia led to the premature differentiation of Tbx18-positive epicardial cells to CoSMCs *in vivo*. (**A,B**) Analysis of the differentiation of Tbx18-positive epicardial cells to CoSMCs by double immunofluorescence for YFP (green) and markers of CoSMCs (red, α-SMA and myh11) in E16.5 and neonatal heart sections from Tbx18:Cre/R26R^EYFP^ mice. Nuclei were co-stained with DAPI. Scale bar is 50 μm. (**C**) β-gal staining of E18.5 heart sections from Tbx18:Cre/R26RLacz mice showed that Tbx18 was expressed in a subset of CoSMCs. Scale bar is 100 μm. Rectangles in the upper right corners of panels (**A,B**) represent the high-magnification views of the areas indicated by arrows. (**D**) Hypoxia intervention for 24 h resulted in the premature expression of CoSMCs markers in the epicardium, which merged with YFP fluorescence in E14.5 Tbx18:Cre/R26R^EYFP^ hearts. Scale bar is 50 μm. (**E**) CoSMCs markers were not expressed in the epicardium of E14.5 Tbx18:Cre/R26R^YFP^ hearts that developed under normoxia. DAPI staining indicated the nuclei. Arrows indicated CoSMCs marker-positive cells in the E14.5 epicardium of Tbx18:Cre/R26R^EYFP^ hearts. Scale bar is 50 μm.

To investigate the differentiation of Tbx18-positive epicardial cells to CoSMCs *in vivo*, we isolated Tbx18:Cre/R26R^EYFP/LacZ^ tracing embryos and observed YFP or LacZ expression in these embryos. Immunofluorescence showed that the CoSMC-specific markers α-SMA and myh11 merged with YFP fluorescence in sections of E16.5 and neonatal Tbx18:Cre/ R26R^EYFP^ mice (Fig. 4A,B). Analysis of β-gal staining in E18.5 Tbx18:Cre/R26R^LacZ^ embryos also showed that a subset of CoSMCs was positive for β-gal staining (Fig. 4C). Collectively, these results indicated that a subset of Tbx18-positive epicardial cells eventually differentiated into CoSMCs after their migration into the myocardial wall.

### Hypoxia led to the premature differentiation of Tbx18-positive epicardial cells to CoSMCs *in vivo*

During normal development, Tbx18-positive epicardial cells do not differentiate into CoSMCs until they reach their final destination surrounding the coronary vessels. Immunofluorescence results showed that CoSMC markers were not expressed in the epicardium of Tbx18:Cre/R26R^EYFP^ embryos at E14.5 (Fig. 4E). To explore the effects of hypoxia on the differentiation of Tbx18-positive epicardial cells *in vivo*, we constructed a hypoxia model of the embryonic epicardium at E14.5. CoSMC-specific markers were prematurely expressed and merged with YFP fluorescence in the epicardium of E14.5 Tbx18:Cre/R26R^EYFP^ hearts (Fig. 4D). This result suggested that hypoxia led to the premature *in situ* differentiation of Tbx18-positive epicardial cells to CoSMCs.

### Hypoxia was sufficient to induce the expression of Snail in Tbx18-positive epicardial cells *in vivo*

**Figure 5:**
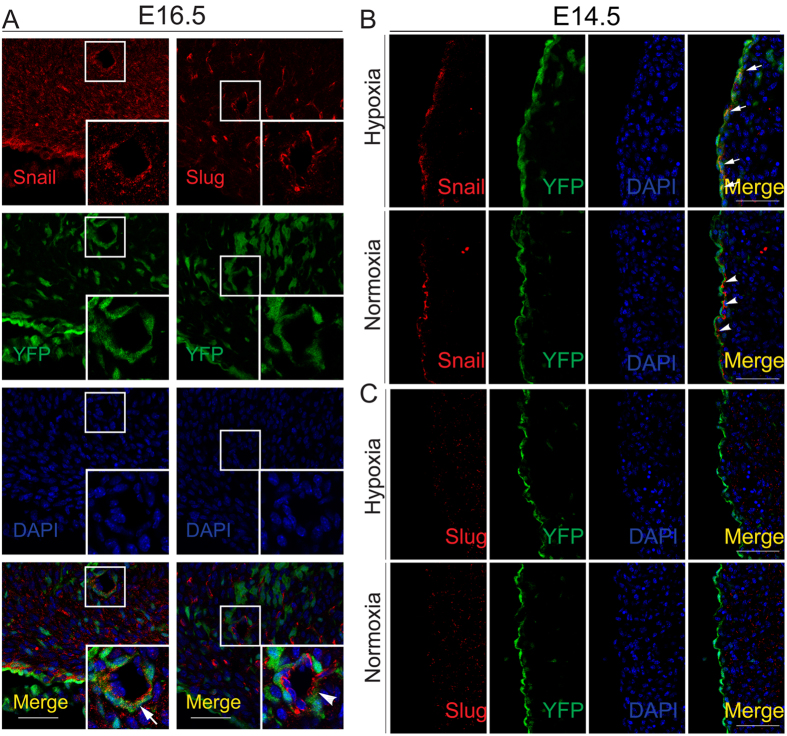
Expression of major EMT regulators in Tbx18-derived coronary vessels and Tbx18-positive epicardium. (**A**) Immunofluorescence for Snail and Slug in Tbx18-positive epicardial-derived CoSMCs of Tbx18:Cre/R26R^EYFP^ hearts at E16.5. YFP fluorescence is shown in the Tbx18-positive epicardial-derived cells of Tbx18:Cre/R26R^EYFP^ mice. Panels in the lower right corner of (**A**) represent higher magnification views of the boxed areas in (**A**) Arrow indicates the merge of Snail with YFP. Arrowhead indicates that Slug did not merge with YFP. Scale bar is 50 μm. (**B,C**) Immunofluorescence analyses of the Tbx18-lineage reporter YFP and Snail/Slug in E14.5 Tbx18:Cre/R26R^EYFP^ embryos after 24 h of maternal hypoxia or normoxia. Arrows and arrowheads indicate the merge of Snail with YFP in the Tbx18-positive epicardium. Scale bar is 50 μm.

We further explored the effects of hypoxia on the primary regulators of EMT *in vivo* with the embryonic epicardial hypoxia model. First, immunofluorescence showed that a subset of CoSMCs derived from Tbx18-positive epicardial cells expressed Snail, rather than Slug, in the hearts of E16.5 Tbx18:Cre/R26R^EYFP^ mice (Fig. 5A). We then investigated the expression of Snail and Slug in the epicardium of E14.5 Tbx18:Cre/R26R26^EYFP^ mice after hypoxia intervention. As shown in Fig. 5B,C and Supplementary Fig. S11, a minority of Tbx18-positive epicardial cells were observed to be Snail-positive at E14.5. However, after 24 h of hypoxia intervention, the expression of Snail was markedly increased and merged with YFP fluorescence in the epicardium, compared with the normal developing heart. Meanwhile, when the hypoxia duration was prolonged to 36 h, Snail was still obviously expressed in the epicardium (Supplementary Fig. S11). However, Slug did not merge with YFP fluorescence in the epicardium, with or without hypoxia intervention (Fig. 5C). This result suggested that the expression of Slug in the epicardium was not related to hypoxia. Hypoxia was sufficient to induce the expression of Snail in Tbx18-positive epicardial cells *in vivo*.

### Hypoxia intervention did not affect the migration of Tbx18-positive epicardial cells into the myocardium *in vivo*

**Figure 6:**
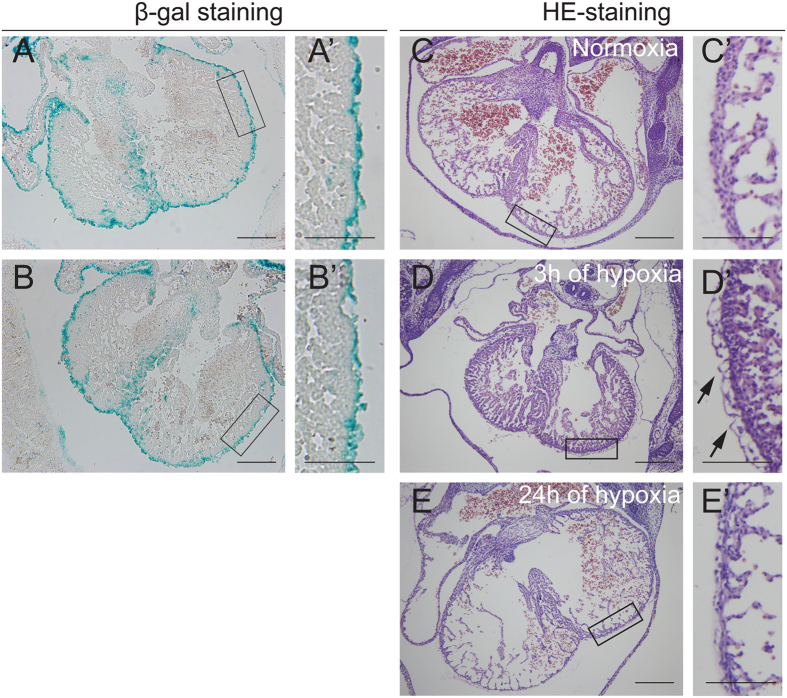
Changes in the migration and detachment of the epicardium after hypoxia intervention at E14.5. (**A,B**) For the migration analysis, β-gal staining was performed in whole hearts of E14.5 Tbx18:Cre/R26R^Lacz^ mice exposed to normoxia (**A**) or 24 h of maternal hypoxia (**B**). (**C–E**) HE stained sections of hearts exposed to normoxia, 3 h of hypoxia, and 24 h of hypoxia. Panels in (A’–E’) represent higher magnification views of the boxed areas in (**A–E**). Arrows in D’ indicate the detached epicardium. Scale bars in (**A–E**) are 500 μm. Scale bars in A’ and B’ are 50 μm and scale bars in (C’–E’) are 100 μm.

We investigated the effects of hypoxia on the migration of Tbx18-positive epicardial cells *in vivo* via β-gal staining of E14.5 Tbx18:Cre/R26R^LacZ^ hearts. Tbx18-positive epicardial and EPDCs were detected in the epicardium, subepicardium and interventricular septum, with some cells also observed in the free myocardial wall arranged in parallel clusters (Fig. 6A,A’). After 24 h of hypoxia intervention, β-gal staining showed no obvious difference in the distribution of these cells between hypoxia- and normoxia-exposed hearts (Fig. 6B,B’). Thus, the migration of Tbx18-positive epicardial cells was not significantly affected by hypoxia at E14.5 *in vivo*. We also analysed E14.5 hearts with HE staining after different durations of hypoxia intervention. A smooth-surfaced epicardium was observed in E14.5 hearts under normal conditions (Fig. 6C,C’). After 3 h of hypoxia, the epicardium appeared irregular and ruffled with focal regions that were unattached to the myocardium (Fig. 6D,D’). Meanwhile, normoxia for 3 h did not induce ruffled detachment of the epicardium from myocardium (Supplementary Fig. S12). However, after 24 h of hypoxia, epicardial cells were subsequently attached to the myocardium (Fig. 6E,E’). These results indicated that the epicardium was sensitive to hypoxia, which temporarily delayed the adhesion of epicardial cells to the myocardium. Thus, *in vivo* hypoxia intervention did not affect the migration of Tbx18-positive epicardial cells into the myocardium but did lead to temporary epicardial detachment.

## Discussion

We cultured Tbx18-positive epicardial cells based on YFP fluorescence and β-gal staining of Tbx18:Cre/R26R^EYFP/LacZ^ tracing mice. With these fate-mapping models, we found that *in vivo* and *in vitro* hypoxia intervention induced CoSMCs differentiation and the EMT progression of Tbx18-positive epicardial cells. Meanwhile, Snail was likely to be a downstream target of HIF-1α during hypoxia-induced CoSMCs differentiation.

Tbx18-positive epicardial cells were previously established to be progenitor cells with the potential to differentiate to CoSMCs, fibroblasts, and sinoatrial node cells^7^,^8^,^9^,^10^. Using Tbx18:Cre/R26R^EYFP /Lacz^ fate-mapping mouse models, we were able to visualize the expression of endogenous Tbx18 during embryonic heart development^9^. These two fate-mapping models are reliable methods to observe the fates of Tbx18-positive cardiac progenitor cells *in vivo* and *in vitro*. To isolate Tbx18-positive epicardial cells, we first genotyped the Tbx18:Cre/R26R^EYFP/LacZ^ tracing mice and then cultured the cells from E11.5 tracing hearts. Using this method, we successfully screened for Tbx18-positive epicardial cell populations and could trace their differentiation according to their YFP fluorescence and β-gal staining. In addition, the cells obtained by this method were highly pure. Meanwhile, when cultured *in vitro*, these cells were similar in terms of their shape and arrangement to the cells on the heart surface *in vivo*, which was conducive to studying the mechanism underlying the EMT of Tbx18-positive epicardial cells.

Early embryonic cardiovascular development occurs in a hypoxic environment^22^,^23^. Under hypoxia, the degradation of HIF-1α was markedly decreased, and HIF-1α was able to enter the nucleus and combine with HIF-1β^32^,^33^. This combination formed active HIF-1, which could further bind to HRE fragments in target genes to promote their expression^34^,^35^. CoSMCs are an important part of coronary vessel formation, and it is of great importance to elucidate the origin and differentiation mechanisms of CoSMCs. The results of our study suggested that hypoxia signal is one of the mechanisms that induces Tbx18-positive epicardial cells to differentiate into CoSMCs. CoCl_2_ was used in our study to induce hypoxic conditions in the cells. CoCl_2_ is an ionic antagonist that replaces the Fe^2+^ in PHD and inhibits cellular oxygen absorptivity under normoxia; a hypoxic state is then subsequently formed^28^,^29^. Our results showed that CoCl_2_-induced hypoxia successfully induced the differentiation of Tbx18-positive epicardial cells to CoSMCs *in vitro* and that the differentiation rate reached nearly 60%. After blocking HIF-1α with 2ME2, the differentiation rate was significantly decreased. This result suggested that hypoxia-induced differentiation was primarily mediated by the regulation of HIF-1α. We also observed the differentiation of Tbx18-positive epicardial cells under normoxia, but the proportion was very small, with only 3.7% ± 1.95% of cells differentiated after 72 h of culture.

To explore the effects of hypoxia on the differentiation of Tbx18-positive epicardial cells *in vivo*, we constructed an E14.5 epicardial hypoxia model through the sustained inhalation of 15% oxygen by pregnant mice. Previously, this method was mainly applied to the research of embryonic heart development in poultry by cultivating eggs for a specific period of time in a hypoxia incubator^36^. Marie *et al*. confirmed that hypoxia could be induced in the hearts of embryonic mice by the persistent inhalation of low oxygen concentrations by pregnant female mice^37^. We adopted 15% O_2_ to induce hypoxia in the E14.5 embryonic epicardium. With prolonged inhalation time, the epicardium of E14.5 fetuses continued to be hypoxic. After 24 h of inhalation of 15% O_2_ in pregnant mice, the hypoxic areas extended from the epicardium to the myocardium of E14.5 fetuses. During our induction of embryonic epicardial hypoxia, we observed the epicardium temporarily detaching from the heart at the beginning of hypoxia induction but did not observe obvious pathological changes, such as heart failure, myocardial apoptosis or myocardial thinning. With this model, we observed a significant increase in the expression of HIF-1α in Tbx18-positive epicardial cells and the premature differentiation of Tbx18-positive epicardial cells to CoSMCs in the epicardium of E14.5 hearts.

EMT is a phenomenon involving the transition of epithelial cells into mesenchymal cells during embryonic development in terms of cell structure and function^38^. The differentiation of epicardial cells to CoSMCs is primarily mediated by the EMT process^2^,^3^,^4^. The disappearance of the epithelial phenotype represents the beginning of the EMT process in cells. Hypoxia intervention caused ZO-1 to disappear from the Tbx18-positive epicardial cell-cell junctions and markedly decreased the expression of epicardial markers (E-cadherin and claudin-1). Thus, these data indicate that the EMT process in Tbx18-positive epicardial cells was induced by hypoxia intervention.

Snail and Slug have been established as the main regulators of EMT^14^,^15^,^16^,^17^. Recent studies have shown that the promoter of the Snail gene contains an HRE fragment that could be combined with HIF-1 α in the cellular nucleus to promote EMT progress under hypoxia^27^. Our results showed that the expression of Snail was significantly increased after *in vitro* hypoxia intervention. Meanwhile, hypoxia also caused the increased expression of Snail *in vivo* in Tbx18-positive epicardial cells on the surface of E14.5 hearts. With prior inhibition of HIF-1α, the hypoxia-induced increase in Snail expression was inhibited. Thus, our results indicated that hypoxia regulated the EMT process in Tbx18-positive cells and Snail was a potential downstream target of HIF-1α. Meanwhile, the knockdown of Snail also significantly decreased the expression of α-SMA and myh11 in these hypoxic cells, which suggested that Snail played a key role in hypoxia-induced differentiation.

Hypoxia intervention and mesenchymal transition have been reported to increase cell motility^27^,^31^. We observed that hypoxia promoted the motility of Tbx18-positive epicardial cells *in vitro*. However, the *in vivo* migration of these cells into the myocardium was not significantly altered after 24 h of hypoxia intervention in the E14.5 heart. The temporary detachment of the epicardium was observed after 3 h of hypoxia intervention *in vivo*. This finding may represent the anatomical factor that inhibits the migration of cells to the myocardium. Meanwhile, hypoxia promoted the premature differentiation of CoSMCs in the E14.5 epicardium in our study. Thus, while hypoxia could promote the motility of Tbx18-positive epicardial cells, temporary epicardial detachment and premature differentiation likely suppressed the migration of epicardial cells to the internal myocardium. Thus, the *in vivo* migration of Tbx18-positive cells was not significantly affected by hypoxia intervention.

## Methods

### Mice

Animals experiments were performed under protocols approved by the animal research committee of Chongqing Medical University. All animal experiments were carried out in accordance with relevant guidelines and regulations of the animal research committee of Chongqing Medical University. Our laboratory has bred Tbx18:Cre knock-in mice (Evans laboratory) and the Cre-lineage reporter R26R^EYFP^ and R26R^Lacz^ mice (Jackson Laboratory). These three mouse lines were maintained on a C57BL/6 background. To generate Tbx18:Cre/R26R^EYFP/LacZ^ double heterozygous mice, we crossed the Tbx18:Cre knock-in mice with the R26R^EYFP^ mice or R26R^LacZ^ mice separately and then screened out these double heterozygous mice by the PCR method, according to the reported literature^39^,^40^. Using these mice, we were able to visualize the expression of endogenous Tbx18 during embryonic heart development^9^.

### β-galactosidase staining

In our research, the β-galactosidase staining of embryonic heart sections and cultured cells was performed as previously described^41^. β-gal staining of the Tbx18-positive cells in Tbx18:Cre/R26R^LacZ^ double heterozygous embryos was positive, and these cells exhibited dark blue staining.

### Isolation and culture of Tbx18-positive epicardial cells

The isolation and culture methods for primary epicardial cells were previously reported in the literature^12^,^18^. Our research incorporated some modifications of these protocols and cultured Tbx18-positive epicardial cells with the Cre-LoxP lineage tracing technique. To generate Tbx18:Cre/R26R^EYFP/LacZ^ tracing Tbx18-positive epicardial cells, Tbx18:Cre knock-in mice were crossed with the lineage reporter R26R^EYFP^ and R26R^Lacz^ mice separately. Embryonic hearts from E11.5 Tbx18:Cre/R26R^EYFP/LacZ^ mice were dissected, placed in 1% gelatin-coated 6-well plates and directly covered with sterile coverslips. The hearts were cultured in serum-free DMEM containing 100 μg/mL streptomycin and 100 units/mL penicillin and incubated at 37 °C. After 48 h, the epicardial cells had migrated onto the dish and formed an epithelial monolayer. Then, we removed the hearts and coverslips and changed the medium. The Tbx18-positive epicardial cells were further cultured in DMEM containing 10% fetal calf serum, which was replaced every other day.

### Quantitative reverse transcription PCR (qRT-PCR)

Total RNA was first extracted from the cells with Trizol (Takara), according to the manufacturer’s protocol. Using the PrimeScript Reverse Transcriptase reagent kit (Takara), 1 μg of total RNA was then reverse-transcribed into cDNA. qRT-PCR was performed on a C1000 thermal cycler (BioRad) with the following cycle conditions: 95 °C for 30 s, followed by 40 cycles of 95 °C for 5 s, 60 °C for 30 s, and 72 °C for 30 s. SYBR premix Ex Taq (TaKaRa) was used in each reaction. The internal control used in our study was GAPDH. We determined the relative gene expression using the 2 -ΔΔCT method and normalized the gene expression levels to GAPDH levels. The primer sequences are provided in Supplementary Table S1.

### Knockdown of Snail in hypoxic Tbx18-positive epicardial cells

Tbx18-positive epicardial cells were transfected with Snail siRNA (siSnail; MSS277145, Invitrogen) or control siRNA (siControl; SIC-001, Sigma), using Lipofectamine RNAiMAX (Invitrogen) according to the manufacturer’s instructions. Afterwards, the transfected cells were cultured in DMEM with 200 μmmol/L CoCl_2_. The experiment was grouped as follows: (1) Hypoxia group: non-transfected Tbx18-positive epicardial cells were cultured with 200 μmmol/L CoCl_2_; (2) Hypoxia + siSnail group: Tbx18-positive epicardial cells transfected with Snail siRNA were cultured with 200 μmmol/L CoCl_2_; (3) Hypoxia + siControl group: Tbx18-positive epicardial cells transfected with control siRNA were cultured with 200 μmmol/L CoCl_2_. After 48 h of culture, the cells in each group were harvested for qRT-PCR and Western blot analysis.

### Immunofluorescence

Primary epicardial cells cultured on coverslips were first fixed in 4% paraformaldehyde (PFA) at room temperature for 15 min. Next, 0.25% Triton was used to permeabilize the cells for 10 min at 37 °C. Treatment with 10% goat serum for 30 min at 37 °C was used to block non-specific binding, and the cells were subsequently incubated with primary antibodies directed against Tbx18 (1:100, Abcam), ZO-1 (1:100, Abcam), HIF-1α (1:50, Abcam), Myh11 (1:300, Abcam), α-SMA (1:200, Abcam), Snail (1:100, Abcam), or Slug (1:100, Abcam) at 4 °C for 24 h. We then incubated the cells with Cy3-conjugated goat anti-rabbit IgG (CWBIO) for 45 min at 37 °C and stained the nuclei with 4′, 6-diamidino-2-phenylindole (DAPI) for 6 min at room temperature.

For frozen sectioning, mouse embryos were isolated, fixed in 4% PFA for 4–6 h at 4 °C, dehydrated using 30% sucrose for 24 h at 4 °C and then embedded in OCT. We sectioned the entire embryos into 10-μm-thick sections, incubated the sections with primary and secondary antibodies and stained the nuclei with DAPI, as described above. The stained cells and sections were photographed using a Nikon Eclipse E600 epifluorescence microscope.

### Western blot assay

After washing with PBS, the cells were lysed with RIPA buffer (Beyotime). The supernatants of the cell lysates were collected after centrifugation and quantified with the BCA protein assay. Equal amounts of proteins were separated on SDS-PAGE and transferred onto nitrocellulose membranes. The membranes were blocked with 5% non-fat milk buffer for 1 h at room temperature and incubated with primary antibodies at 4 °C overnight. Then, the membranes were washed and incubated with horseradish peroxidase-conjugated secondary antibody (goat anti-rabbit IgG, CWBIO) for 1 h at room temperature. The membranes were then visualized with enhanced chemiluminescence (ECL, Advansto). GAPDH was used as an internal control for protein loading. The target protein’s relative expression was shown as a ratio of target protein/GAPDH.

### Cell migration assays

Transwell chambers were used to perform migration assays based on the manufacturer’s instructions (Millipore). Cells (2.0 × 10^4^) were seeded into each chamber insert in FBS-free DMEM, and DMEM containing 10% FBS was added to the lower chambers. Each group of cells was then treated with CoCl_2_ or 2ME2 according to the experimental design. The plates were incubated at 37 °C for 24 h. Finally, we removed the cells from the upper chambers with cotton swabs, stained the cells that migrated under of the membrane with crystal violet and counted the cell numbers under a microscope.

### Contractility assay

Agonist-induced contractility of the differentiated cells was assayed as reported^42^. The Tbx18-positive epicardial cells were cultured under CoCl_2_-induced hypoxia for 72 h, washed with PBS, and then stimulated with 1 mmol/L carbachol for 5 min. A Nikon microscope was used to monitor cell contraction and image the same field before and after carbachol treatment.

### Collagen gel contraction assay

Collagen gel contraction assay was performed as previously described with some modification^43^. The cells cultured in normoxia, hypoxia and hypoxia + siSnail conditions were trypsinized and resuspended in DMEM, respectively. Subsequently, collagen gel solution was prepared by mixing collagen type I (Solarbio) with 10 × PBS, pH 7.0. The cell suspension was then added to collagen gel solution on ice to achieve a final concentration of 1 × 10^5^ cells/mL and 1 mg/mL of collagen type I. 500 μl of cell-collagen gel mixture was pipetted into 24-well plates and polymerized at 37 °C for 30 minutes. Thereafter, the gels were mechanically detached from the plate by adding 1 ml serum-free DMEM and then cultured for 6 h. Subsequently, 1 mmol/L carbachol was added to initiate contraction for 30 minutes, and DMEM without carbachol was used as a control. The images were then acquired with a digital camera, and the area of gel was determined by using Image J. Relative gel area was obtained by dividing the final area by the initial area of the gel.

### Construction of the hypoxia model of the E14.5 embryonic epicardium

Our study chose 15% O_2_ to induce hypoxia in the embryonic epicardium, and we observed the hypoxia of the E14.5 fetal epicardium using Hypoxyprobe-1. Detailed information is provided in the Supplementary Methods online.

### Statistical analysis

Each experiment was repeated three times, and the values were expressed as the means ± SD. Student’s t test was performed to compare the differences between two groups. The differences between more than two groups were compared with one-way ANOVA followed by post hoc analysis with Tukey’s test (equal variances) or Welch’s ANOVA test followed by Dunnett’s T3 test (unequal variances). All data were analysed with SPSS 20.0, and P values < 0.05 were considered statistically significant.

## Additional Information

**How to cite this article**: Jing, X. *et al*. Hypoxia induced the differentiation of Tbx18-positive epicardial cells to CoSMCs. *Sci. Rep.* **6**, 30468; doi: 10.1038/srep30468 (2016).

## Supplementary Material

Supplementary Information
