# Supplementary material for: Hypoxia induced the differentiation of Tbx18-positive epicardial cells to CoSMCs
Source: Sci Rep. 2016 Jul 26;6:30468. doi: 10.1038/srep30468 (PMC4960593; doi:10.1038/srep30468)
Supplement: Supplementary Information [file srep30468-s1.pdf]

Hypoxia induced the differentiation of Tbx18-positive epicardial cells to CoSMCs

Xiaodong Jing [1], Yulin Gao [1], Songlin Xiao [1], Qin Qin [1], Xiaoming Wei [1], Yuling Yan [1], Ling Wu [1], Songbai Deng [1], Jianlin Du [1], Yajie Liu [1], Qiang She [1, *]

[1] Department of Cardiology, The Second Affiliated Hospital of Chongqing Medical University, Chongqing, 400016, China.

* Correspondence: E-mail: qshe98@hotmail.com

Supplementary information

Supplementary methods

Cell survival assay and the screening of drug concentrations

Five thousand Tbx18-positive epicardial cells were plated into each well of a 96-well plate and cultured for 24 h in a 37 ℃ incubator. Then, different concentrations of $CoCl_2$ or 2ME2 were used to treat the cells. At the indicated time points (24, 48, or 72 h), 20 μl of CCK-8 solution was added to each well, and the plates were then incubated for another 1 h at 37 ℃. Finally, we measured the absorbance at 450 nm with a spectrophotometer (Bio-Rad, Hercules, CA, USA).

It was previously confirmed that a certain range of concentrations of $CoCl_2$ could induce the hypoxic response in cells, but higher concentrations of $CoCl_2$ could reduce cell survival and lead to severe cellular toxicity.[1,2] As shown in Supplementary Fig. S2A online, 0-200 μmol/L $CoCl_2$ did not significantly affect the survival of Tbx18-positive epicardial cells, while 400 μmol/L $CoCl_2$ produced obvious cytotoxicity. After culturing the cells in 200 μmol/L $CoCl_2$ for 24 h, HIF-1α accumulated in the nucleus of Tbx18-positive epicardial cells (Supplementary Fig. S3A online). In addition, the mRNA levels of HIF-1α were increased by approximately 1-fold (Supplementary Fig. S3B online). Thus, 200 μmol/L $CoCl_2$ was chosen to induce the hypoxic response in our study.

2ME2 is an inhibitor of HIF-1α that acts primarily by inhibiting its nuclear accumulation.[3] Thus, to screen for 2ME2 concentrations that effectively block HIF-1α in hypoxia, we cultured the cells in 200 μmol/L $CoCl_2$ with varying concentrations of 2ME2. As shown in Supplementary Fig. 2B online, when combined with 200 μmol/L $CoCl_2$, 0-50 μM 2ME2 did not show obvious cytotoxicity, while 100 μmol/L 2ME2 impacted the cells. Meanwhile, 50 μmol/L 2ME2 fully blocked the nuclear accumulation of HIF-1α without affecting the transcription of HIF-1α mRNA (Supplementary Fig. S3 online). Thus, we selected 50 μmol/L 2ME2 to block HIF-1α in our study.

Construction of the hypoxia model of the E14.5 embryonic epicardium

Previous research reported that hypoxia could be induced in the hearts of embryonic mice by the persistent inhalation of low oxygen concentrations by the pregnant female mice.[4] Our study chose 15% O_2 to induce hypoxia in the embryonic epicardium and observed the hypoxia of E14.5 fetal

epicardium using Hypoxyprobe-1. On gestational day E14.5, pregnant female mice were first placed under normoxia (21% O_2) or 15% O_2 for a specified amount of time (3 h or 24 h). For investigations of fetal tissue hypoxia, the pregnant mice were injected intraperitoneally with 60 mg/kg of pimonidazole (Hypoxyprobe-1 Omni kit; HPI) dissolved in a 0.9% saline solution. Next, the pregnant females were euthanized 1.5 h after the injection. The fetuses were removed, fixed in 4% PFA at 4 ℃ overnight and processed as described above. Pimonidazole binding was detected by an affinity purified rabbit anti-pimonidazole antibody and Cy3-conjugated goat anti-rabbit IgG (CWBIO) as described above.

As shown in Supplementary Fig. S10A online, the E14.5 hearts were not hypoxic under normoxia. However, after 3 h of maternal hypoxia, pimonidazole staining was markedly increased and merged with YFP fluorescence in the E14.5 epicardium. After 24 h, the hypoxic region encompassed the myocardium and ventricular septum. The expression of HIF-1α in the epicardium of E14.5 Tbx18:Cre/R26R^{EYFP} mice was also examined. Under normoxic conditions, a limited number of Tbx18-positive epicardial cells were observed to be HIF-1α-positive at E14.5. However, after 24 h of hypoxia, the number of Tbx18-positive epicardial cells expressing HIF-1α was significantly increased (Supplementary Fig. S10B online). Thus, we successfully constructed an *in vivo* embryonic epicardium hypoxia model.

Supplementary Tables

Table S1. The primer sequences used for qRT-PCR

Gene		Sequence (5'-3')
Tbx18	Forward	TCGATACAGCACATCCTTGG
	Reverse	GCTGCTCACACTCACGTAGG
cTnT	Forward	AGCCCACATGCCTGCTTAAA
	Reverse	TCTGAACAGGGACTGCACAC
HIF-1α	Forward	TGAACATCAAGTCAGCAACG
	Reverse	CACAAATCAGCACCAAGCAC
α-SMA	Forward	GGGAGTAATGGTTGGAATGG
	Reverse	GGTGATGATGCCGTGTTCTA
Myh11	Forward	CAGTGACAAGGTCCACAAGC
	Reverse	CAAGGGAAGCCACATCTTTG
Snail	Forward	CCATTCTCCTGCTCCCACT
	Reverse	CCTGGCACTGGTATCTCTTCA
Slug	Forward	ACAGCGAACTGGACACACAC
	Reverse	GGGTAAAGGAGAGTGGAGTGG
N-cadherin	Forward	GTGGAGAACCCCATTGACAT
	Reverse	CATACGTCCCAGGCTTTGAT
GAPDH	Forward	AAGTTCAACGGCACAGTCAAGG
	Reverse	CGCCAGTAGACTCCACGACATA

Supplementary Figures

Figure S1. Immunofluorescence analysis of the expression of periostin in cultured Tbx18-positive epicardial cells

YFP fluorescence was observed in Tbx18-positive epicardial cells isolated from E11.5 Tbx18:Cre/R26R^{EYFP} hearts. DAPI staining indicates the nuclei. Scale bar is 50 μm.

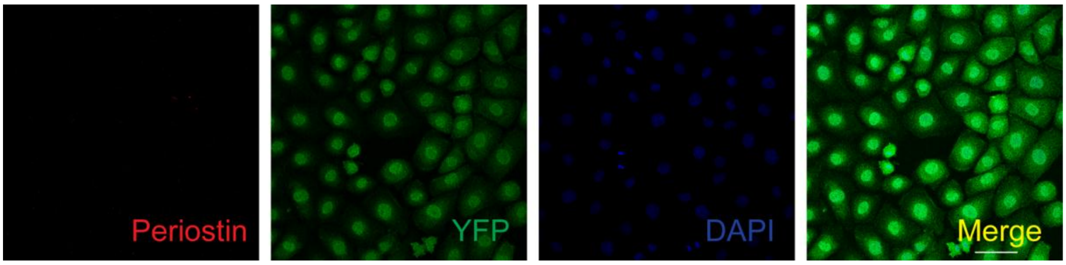

Figure S2. Analysis of cell viability by CCK-8 assays

A: Effect of $CoCl_2$-induced hypoxia on the viability of Tbx18-positive epicardial cells. The data are shown as the mean ± SD of three experiments. *$p < 0.05$, compared with the control group (0 μmol/L) at each time point; one-way ANOVA. B. Effect of 2ME2 in combination with 200 μmol/L CoCl2 on the viability of Tbx18-positive epicardial cells. The cells were treated with 0, 25, 50, 100 μmol/L 2ME2 combined with 200 μmol/L CoCl2 and incubated for 24, 48, or 72 h. The cells cultured with 200 μM CoCl2 served as a positive control. The data are shown as the means ± SD of three experiments. *$p < 0.05$, compared with the positive control group (0 μmol/L) at each time point; one-way ANOVA.

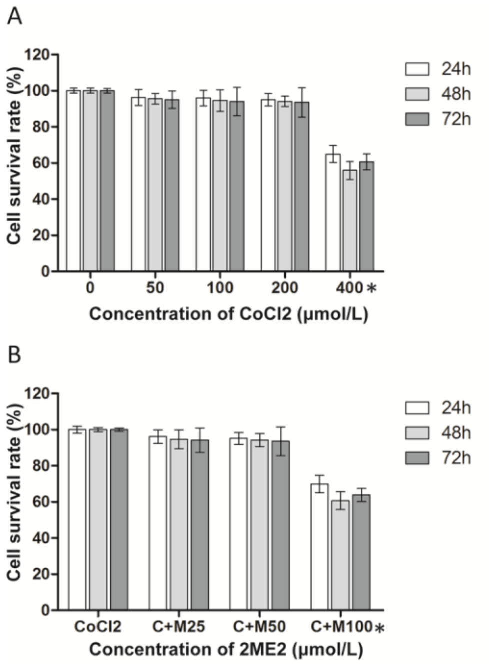

Figure S3. Effect of hypoxia on HIF-1α expression in Tbx18-positive epicardial cells

A: Immunofluorescence analysis of the expression of HIF-1α in Tbx18-positive epicardial cells cultured under hypoxia, hypoxia combined with 2ME2, normoxia, or normoxia combined with 2ME2 for 24 h. YFP fluorescence was shown in Tbx18-positive epicardial cells isolated from Tbx18:Cre/R26R^{EYFP} hearts. DAPI staining indicates the nuclei. Hypoxia was induced by 200 μmol/L $CoCl_2$, and the normoxia combined with 2ME2 group was used as a negative control to exclude the effects of 2ME2 alone on cellular responses. Scale bar is 50 μm. B: The mRNA expression level of HIF-1α was determined in Tbx18-positive epicardial cells cultured under normoxia, hypoxia, hypoxia combined with 2ME2, or normoxia combined with 2ME2 for 24 h, 48 h or 72 h by qRT-PCR. Hypoxia was induced by 200 μmol/L $CoCl_2$. GAPDH was used as an internal control, and the mRNA level of Tbx18-positive epicardial cells cultured under normoxia was used as a control. The data were shown as the mean ± SD of three experiments; *$p < 0.05$, hypoxia group vs. normoxia group at each time point; one-way ANOVA.

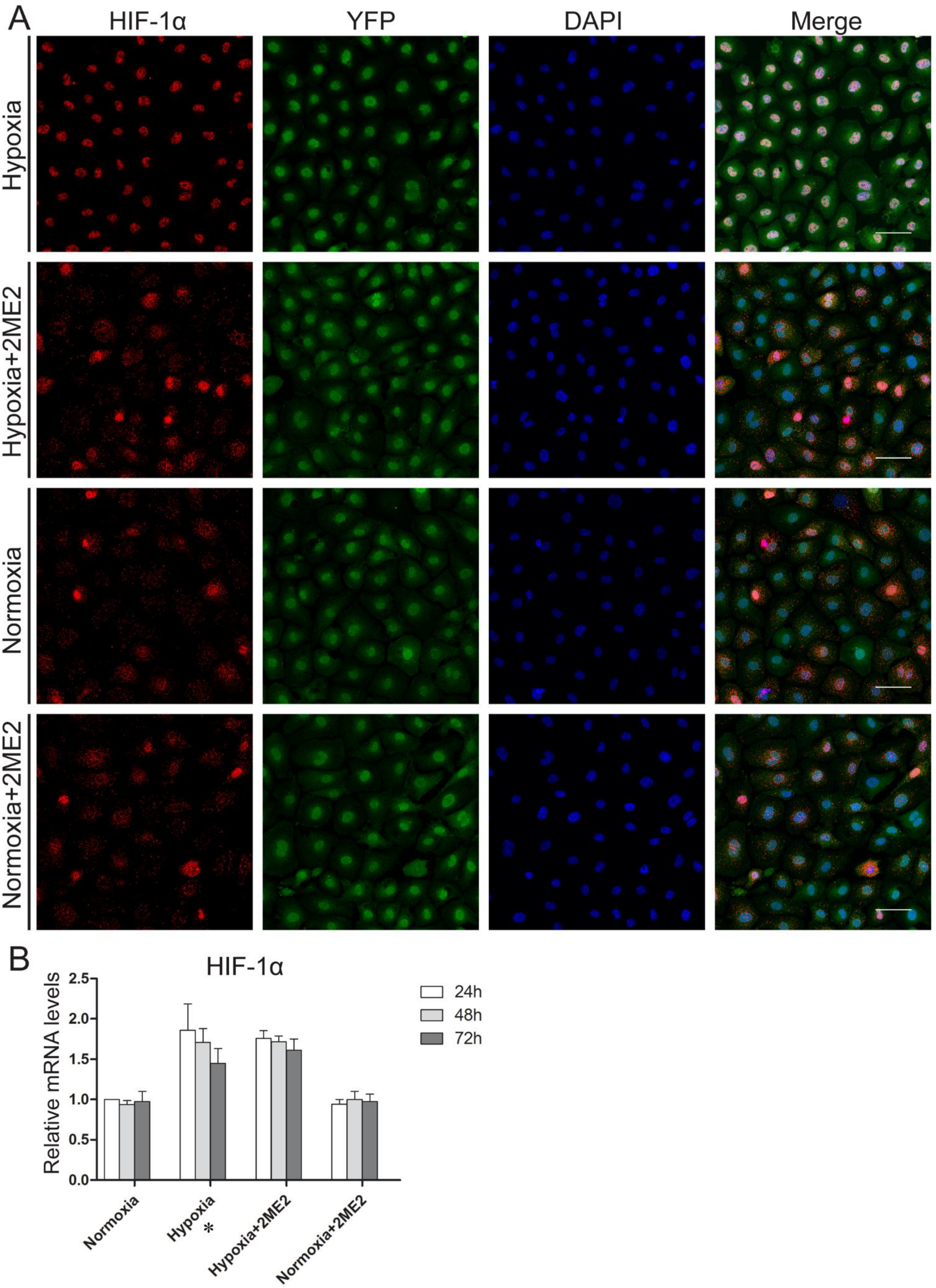
A
HIF-1α
YFP
DAPI
Merge
Hypoxia
Hypoxia+2ME2
Normoxia
Normoxia+2ME2
B
HIF-1α
Relative mRNA levels
2.5
2.0
1.5
1.0
0.5
0.0
24h
48h
72h
Normoxia
Hypoxia
*
Hypoxia+2ME2
Normoxia+2ME2

Figure S4. Carbachol-induced contraction of CoSMCs differentiated from Tbx18-positive epicardial cells

Tbx18-positive epicardial cells were cultured under $CoCl_2$-induced hypoxia for 72 h and then imaged before and after 1 mmol/L carbachol treatment for 5 min. Arrows indicate representative contracted cells. Scale bar is 25 μm.

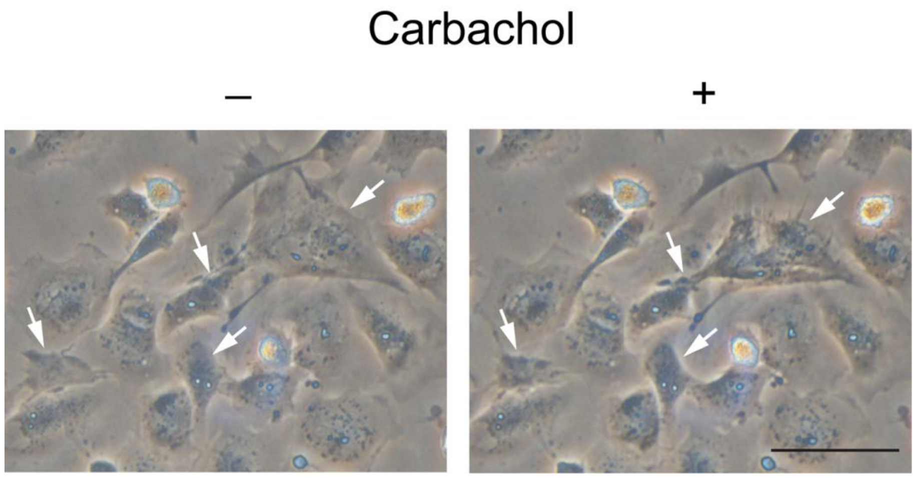

Figure S5. The protein expression of myh11 in hypoxic Tbx18-positive epicardial cells

A: Representative Western blots of myh11 in hypoxia groups or hypoxia + 2ME2 groups at three time points. B: The relative protein expression levels of myh11. GAPDH was used as a loading control, and the protein levels in Tbx18-positive epicardial cells cultured under hypoxia for 24 h were used as controls. The data are shown as the means ± SD of three experiments. *p < 0.05, hypoxia group vs. hypoxia + 2ME2 group at each time point; Student's t test.

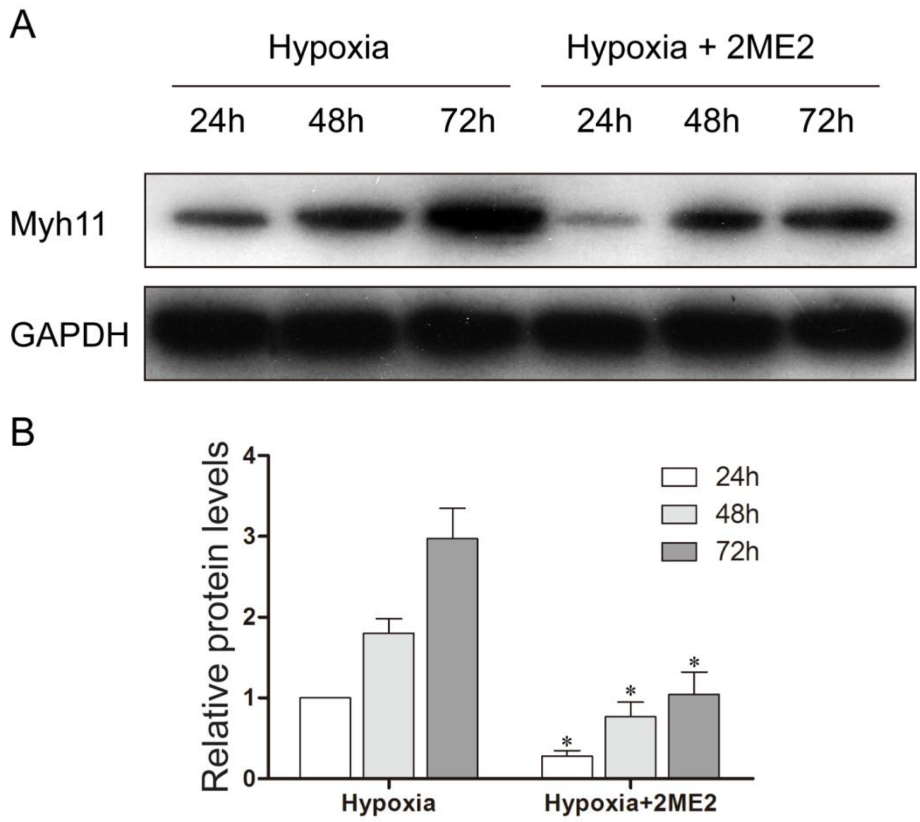

Figure S6. Effect of hypoxia on the protein expression of E-cadherin and claudin-1 in Tbx18-positive epicardial cells

A: Representative Western blots of E-cadherin and claudin-1 in normoxia groups or hypoxia groups at three time points. B: The relative protein expression levels of E-cadherin and claudin-1. GAPDH was used as a loading control, and the protein levels in Tbx18-positive epicardial cells cultured under normoxia for 24 h were used as controls. The data are shown as the means ± SD of three experiments. *p < 0.05, normoxia group vs. hypoxia group at each time point; Student's t test.

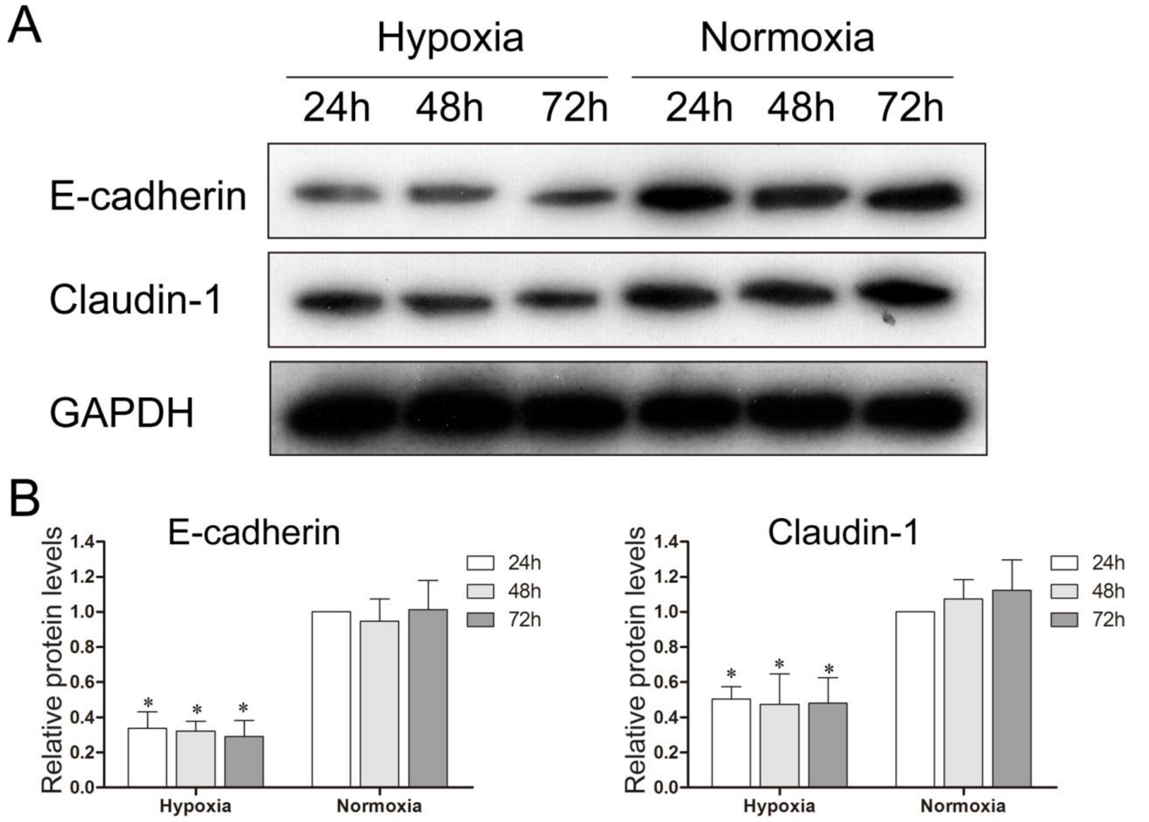

Figure S7. Effect of hypoxia on the mRNA expression of N-cadherin in Tbx18-positive epicardial cells

The mRNA levels of N-cadherin were determined in Tbx18-positive epicardial cells cultured under normoxia and hypoxia at three time points by qRT-PCR. GAPDH was used as an internal control, and the mRNA levels in Tbx18-positive epicardial cells cultured under normoxia were used as controls. The data are shown as the means ± SD of three experiments. *$p < 0.05$, normoxia group vs. hypoxia group at each time point; Student's t test.

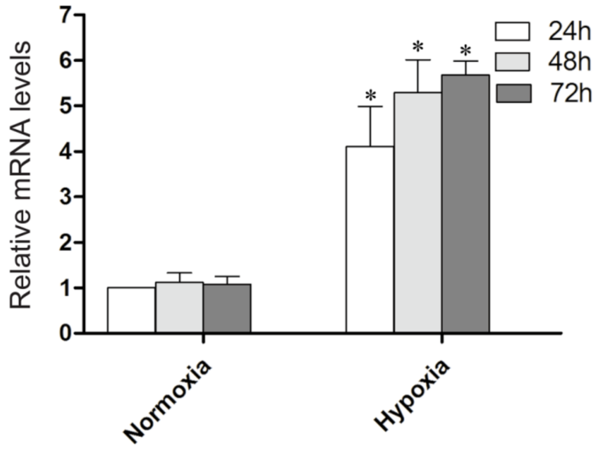

Figure S8. Effects of Snail gene knockdown on hypoxia-induced myh11 and α-SMA expression in Tbx18-positive epicardial cells

Tbx18-positive epicardial cells were transiently transfected with Snail siRNA or control siRNA. The transfected cells were then cultured under $CoCl_2$-induced hypoxia for 48 h. A: The mRNA expression levels of Snail, myh11 and α-SMA were analysed by qRT-PCR after Snail siRNA transfection. GAPDH was used as an internal control, and the mRNA levels of the hypoxia group cells were used as controls. B: The protein expression levels of HIF-1α, myh11 and α-SMA were analysed by Western blot after Snail siRNA transfection. GAPDH was used as a loading control, and the protein levels of the hypoxia group cells were used as controls. The data are shown as the means ± SD of three experiments. *$p < 0.05$, compared with the hypoxia and hypoxia + sicontrol groups; one-way ANOVA followed by Tukey's test.

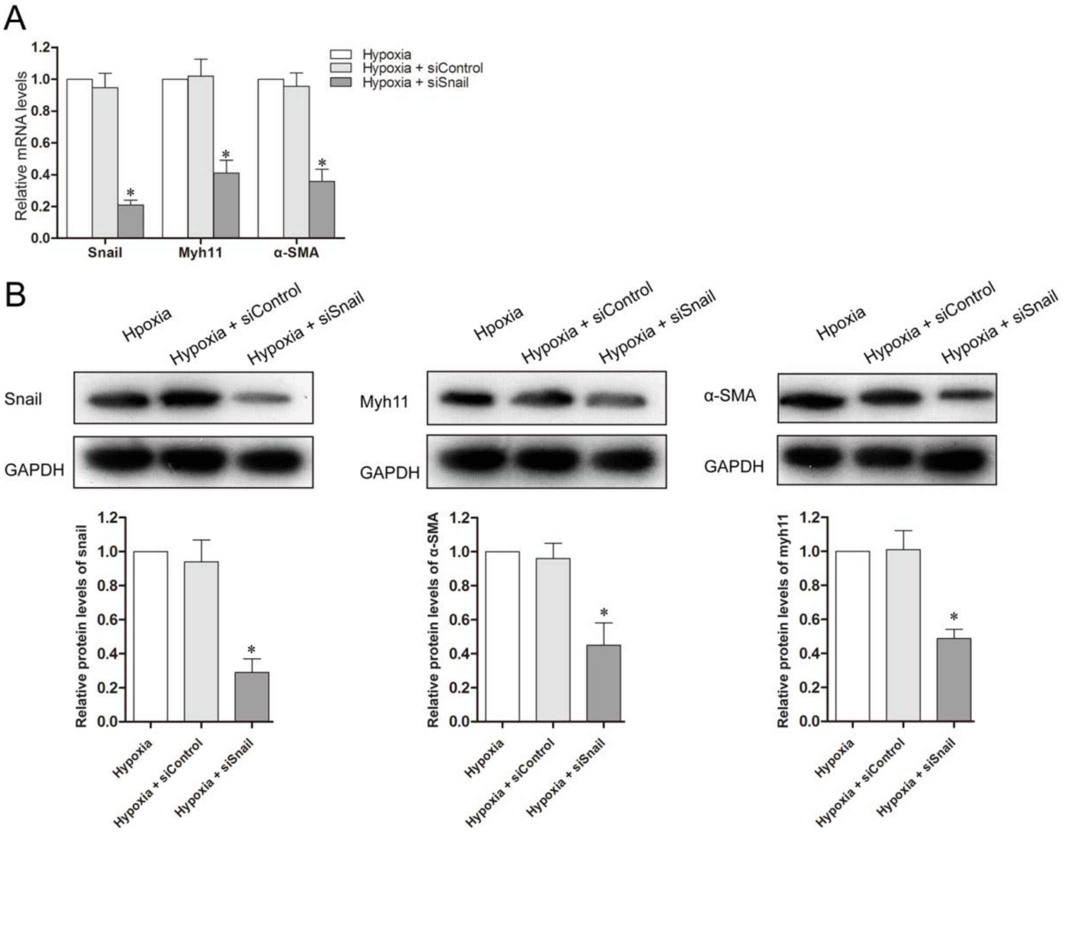

Figure S9. The effect of Snail knockdown on collagen gel contraction.

A: Representative images of collagen gel seeded with Tbx18-positive cells that have been respectively cultured in normoxia, hypoxia and hypoxia + siSnail conditions for 48 h. 1mmol/L carbachol was used to initiate contraction for 30 minutes, and the collagen gel without carbachol treatment was used as a control. B: Quantification of carbachol-mediated gel contraction. Relative gel area was obtained by dividing the final gel area by the initial area of the gel. The data are shown as the means ± SD of three experiments. *p < 0.05, compared with hypoxia control group; Student's t test.

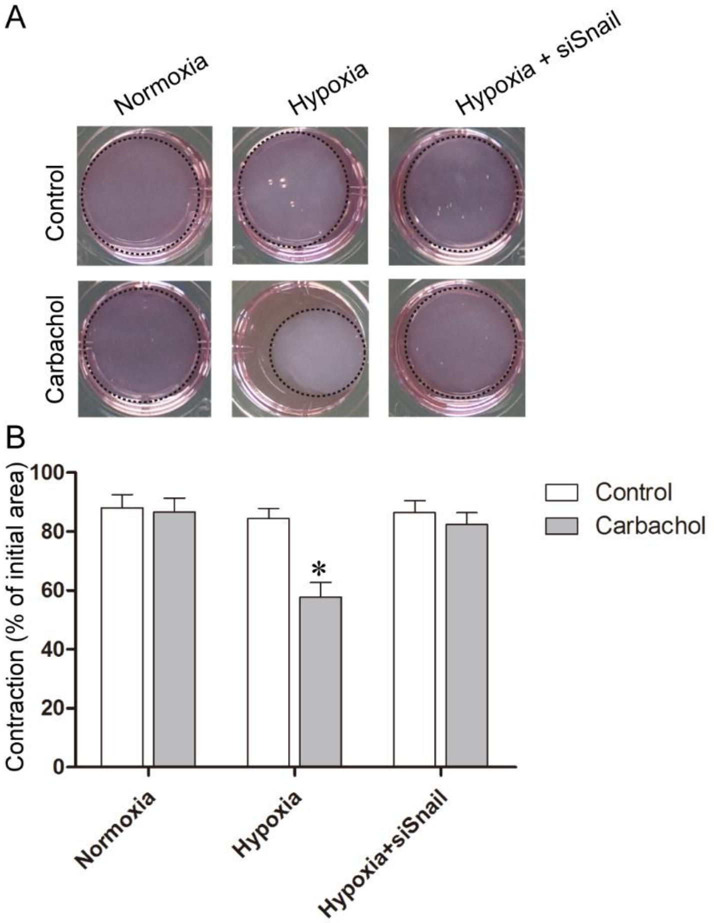

Figure S10. Construction of the hypoxia model of the embryonic epicardium and the assessment of epicardial hypoxia

A: Hypoxyprobe (HPI) was used to detect hypoxia of the E14.5 epicardium after pregnant female mice were placed under normoxia (21% O_2) or 15% O_2 for a specified amount of time (3 h or 24 h). B: Immunofluorescence for the YFP reporter (green) and HIF-1α (red) in sections of E14.5 Tbx18:Cre/R26R^{EYFP} hearts after 24 h of maternal hypoxia and normoxia. Nuclei were counterstained with DAPI. The right panels in Figure A represent higher magnification views of the boxed areas. Scale bar in A is 200 µm. Scale bar in B is 50 µm.

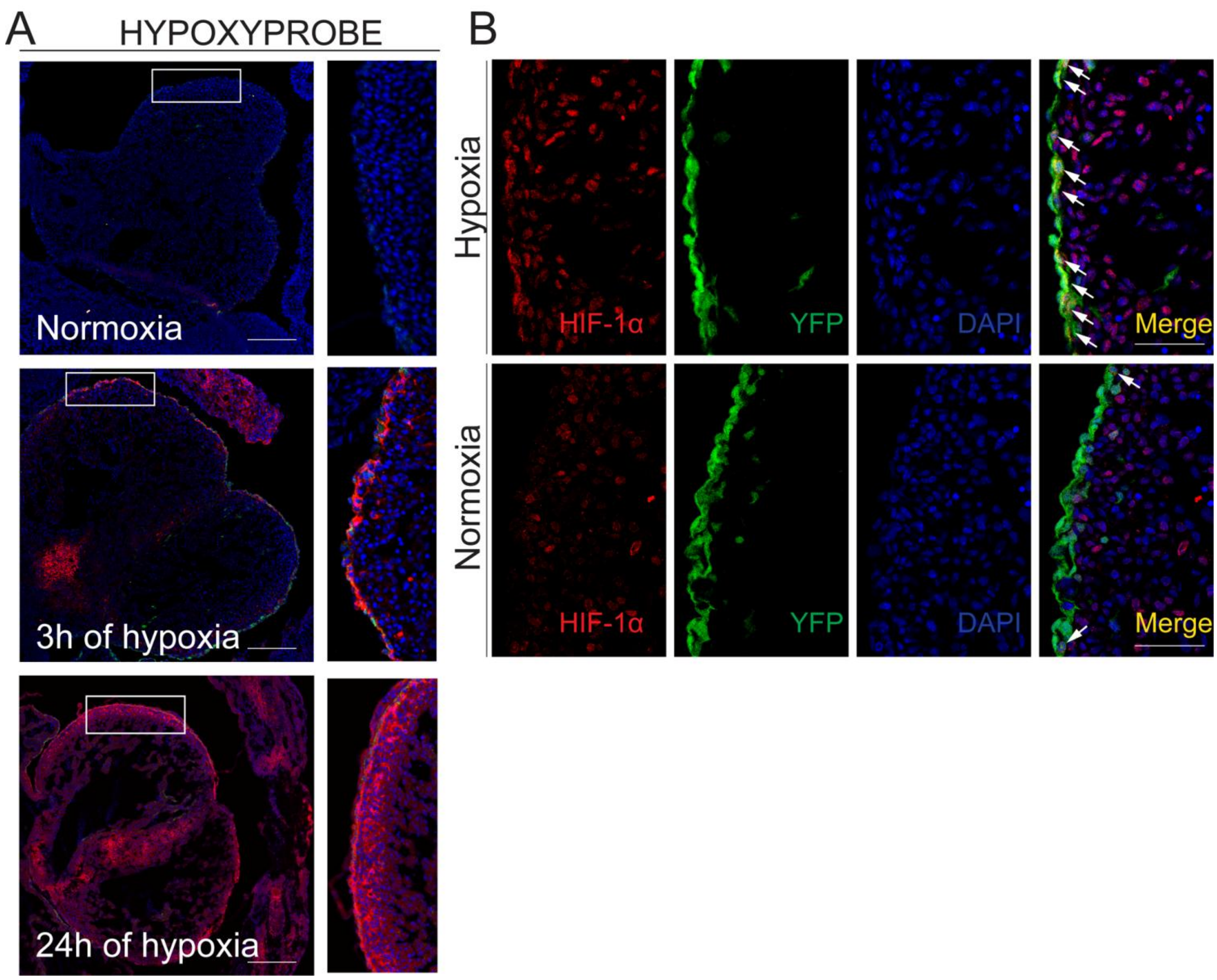

Figure S11. Immunofluorescence staining of Snail in the epicardium of E14.5 Tbx18:Cre/R26R^{EYFP} embryos

Immunofluorescence showed Snail (red) and the YFP reporter (green) in sections of E14.5 Tbx18:Cre/R26R^{EYFP} hearts separately under normoxia, after 24 h of maternal hypoxia and after 36 h of maternal hypoxia. Nuclei were counterstained with DAPI. Scale bar is 100 μm.

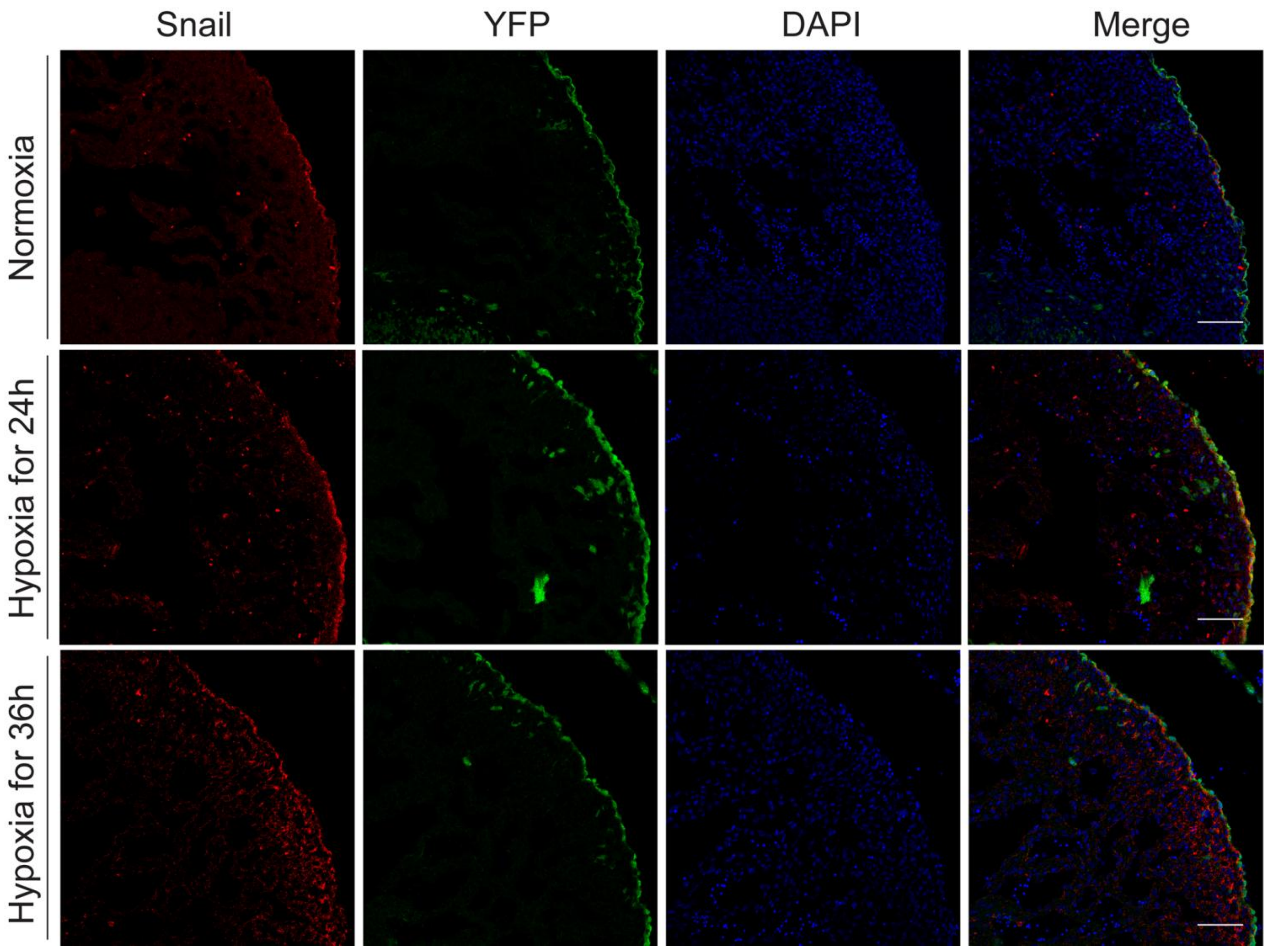

Figure S12. HE staining section of E14.5 heart exposed to 3 h of normoxia.

On gestational day E14.5, pregnant female mice were placed under normoxia for 3 h, and then the heart of embryo was obtained for HE staining. Scale bar is 500 μm.

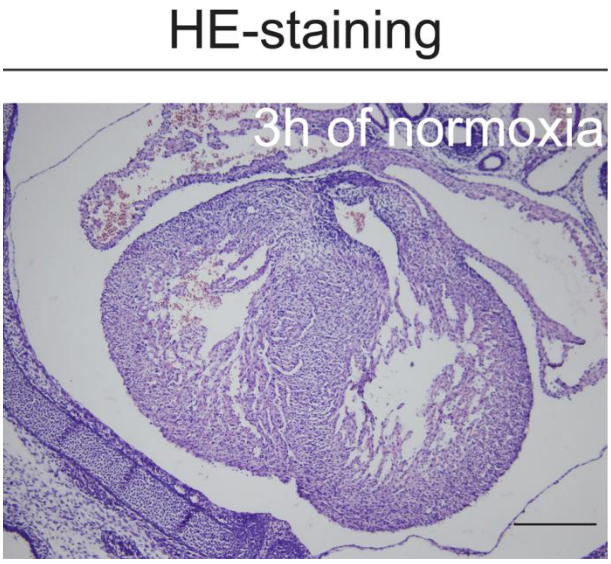

Supplementary References

1. Kong, D. et al. Curcumin inhibits cobalt chloride-induced epithelial-to-mesenchymal transition associated with interference with TGF-beta/Smad signaling in hepatocytes. *Lab Invest.* **95**, 1234-1245 (2015).

2. Ananthula, S. et al. delta-Tocotrienol oxazine derivative antagonizes mammary tumor cell compensatory response to CoCl2-induced hypoxia. *Biomed Res Int.* **2014**, 285752 (2014).

3. Mabjeesh, N. J. *et al.* 2ME2 inhibits tumor growth and angiogenesis by disrupting microtubules and dysregulating HIF. *Cancer cell* **3**, 363-375 (2003).

4. Ream, M., Ray, A. M., Chandra, R. & Chikaraishi, D. M. Early fetal hypoxia leads to growth restriction and myocardial thinning. *Am J Physiol Regul Integr Comp Physiol.* **295**, R583-595 (2008).
